# Supplementary material for: A single amino acid variant in the variable region I of AAV capsid confers liver detargeting
Source: PLoS Pathog. 2025 Sep 17;21(9):e1013533. doi: 10.1371/journal.ppat.1013533 (PMC12456803; doi:10.1371/journal.ppat.1013533)
Supplement: S1 Fig — (a) Schematics showing the barcoded AAV construct, not drawn to scale. The first barcode (bc1) in the TuD and the second barcode (bc2) in the forward PCR primer are rainbow-colored. Note that each unique bc1 was used to pair with a unique capsid variant during rAAV production, and that bc2 was used in PCR to differentiate among tissue samples. (b) The DNA sequence of the construct shown in a. Key elements are color-coded as the scheme shown in a. Gray: ITR. Blue: stuffer. Green: U6 promoter. Yellow: TuD. NNNNNNNN: bc1. (c) Binding sites of the forward and reverse PCR primers shown in a. Binding sites are underlined. Note that bc2 is included at the 5’ end of the forward primer. (PDF) [file ppat.1013533.s001.pdf]

a

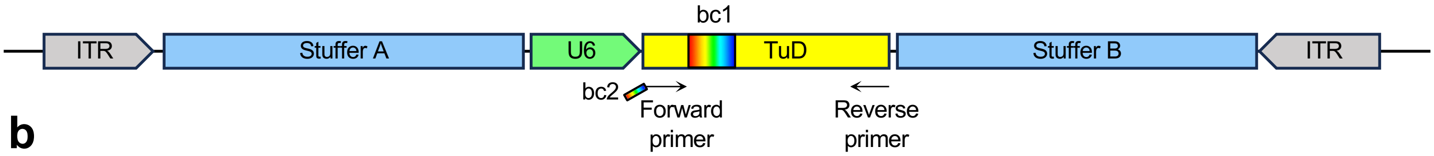

b

ctgcgcgctcgctcgctcactgaggccgcccgggcaaagcccgggcgtcgggcgacctttggtcgcccggcctcagtgagcgagcgagcgcgcagagagggagtggccaactccatcactaggggttccttgtagttaatgat
taacccgccatgctacttatctaccagggtaatggggaagtacaataaaaacaaggctttcaagctgataaaggcttctttcccactcatagaggaaattctttaaagtatccccaaaattgctttcctgaggtgttcctcttggtt
aaccgtctagtgattggggacctttgaaaacactcaggattgaagataatggtcttttctcactattggtgatgtaatcagtgttctgaaaataaaatactattttagctagagcgttatagaagtcagtagaatattcagaatttc
atttgcatttctgatcatcaaggtgaccaccactgccagcatgtgcagggtacctgcattgtgtctcttggccacagcgggtccttctgcaggtggagctgtggtctttgcagttgggcgcagctagcatggcaggtacatgagca
tttggaagagcagtcaggacccatgtgctcagcacatgtgttttcttgggcagttggcagaaaccataagtcttttcccaacttttatgtttgggaagagagagaaagaagccaaatttgctatacgtttgatgttattaaggttgt
catttgaataagtttttaaaaaatgtataacataataactggattgatacaagcatgctgaaagataaactaataactgaacatatccgttaattttttggtatttgtagtgaaaacataattgtaattgaaaacaacattactg
aagagggaaaaatgatttttatgcctcaagcaaaacacacacatatacacaaaaaattaaagacttaagcttttttttttctttccaagaacatctggtttacagtatgctaaaagcaaatttggattaacctagaaattaagtg
tgtgtcatatacaagccattgattaaaccatattaaaatatttagacctcatggaaaggtataaaactctccaccatgtacagatttttattttttgtaaggaaagtaaacaaaactatttcaatgaatcctgtgtcaaaaaaatt
aaaataaatctgtgtgatgtcatctgcgtctgccaacctgcccttccaaagagacgcaagatggaagctttcagtgcttgtcctgaccacccagcaacaggtctttaagcctgaggacagtcattccttacacttttattctccaat
ttctttgctttgtgtaggactttacgggcatctatgttcattcttaaaatatttcgtactaatataaaaatcgtggacctgagtccaagccacaaaccctgaagcaagaatttggtcctgatcttgagtcttctttcacctgcagagcc
ctccccggactctcaggaccataggctgtccaggcactcctgattctgaagccacgggaagtcccaatttcagccattgttggtgagaaacaatccctctttaagagctgcccataagggcaacctgtcccaagaggcttccgat
agcatctgcgtcctcttctgcatgttgtcacccacgaaggaggcctgcgtggcacagagcccacagtgccatatttctgaagcagtggaaggaattgctctgggtgacgccggtggaaggaattgctctccgctgaccctagca
atggatgctgagccaggagcaagtgagaacaaggtcccacgagatcagccggagggcatggggaataagcttctgtcccttcaaggaggccaagtaagataagtttctagttctgtcacattcacagacatctagttcatgac
acagagccttgctttttccaccaggaataaaataccccgatcagcagcttgtgataagttctccccacctgctccctccagcatcccaggagagaacgttaactacacaccttgtttaatgcaaagtcttataatatccatgtgtct
tagaaaataaaattgtagagaccaagaagtgcaaataatcaaattatttaataggaaatttgtgttaggaattccccagtggaaagacgcgcaggcaaaacgcaccacgtgacggagcgtgaccgcgcgccgagcgcgcg
ccaaggtcgggcaggaagagggcctatttcccatgattccttcatatttgcatatacgatacaaggctgttagagagataattagaattaatttgactgtaaacacaaagatattagtacaaaatacgtgacgtagaaagtaat
aatttcttgggtagtttgcagttttaaaattatgttttaaaatggactatcatatgcttaccgtaacttgaaagtatttcgatttcttgggtttatatatcttgtggaaaggacgcgggatccgacggcgctaggatcatcaac**NNN**
NNNNNacgaatctctatatcatcaagtattctggtcacagaatacaacaagccacaacgaatctctatatcatcaagatgatcctagcgccgtctttttttaagctgtcccggatccgacccaggacctaactacgaaagcag
ttttacgtctaaaacacagagatgttttgttactcaagctatacggcatgttgcctttccctgacaaggcagtgtgcctgagaatggatgggatataattattttttttaattccagaaaatttgcttgatgttcataaataatcgatt
caatcactttagcaaaagggaaatgttaaaatgatggaaaattctgttcacctagacttgtctgcagtctttaaatcattctccactccatccctcttgctgacattcggagatgatttcaattaagggcctttgacaaagtactat
agtcatttaaaagatggctttaaagagttcagaaatcccaatagttctccatatcaagagggtgcactataaaattgtaaatgagtgcatgcactggctggggttataaagtgccgttgtttttctggtttgacatttcatggattc
caagtgggcctagcttagtggtgcaacataaatcacggaacggatctggatattgtaaaacttgaatatagaagcattttagaggctgtgaagttaacagtaatcagtaagtattctaatagtatgagttactgctaacattata
attatagagtaaaataataatttttataggcaaggagcactgaaaactaatggtcaggatgattataatagctatgaattaatagatcagttgaaattgtgatgactattttaataatgagactgaaatatcgtttgtgttaaaac
agtgagtctaagagcataatcaaccctcttttgtcaggaagaaacaattccttttaactccaaggtcaccagaatgatcctagagttgtattgtggttactctttctccttggaaatgattcttccgttctcctttgctctgtagagtc
ctattcctctacctcatcaggtaggtctgtgtgtgtgagaatgtttgttctcctttgccctgtagagtcctattcgtctaccttgtcgtgtaggtttgtgtgtgggcgagtatttgttctcctttgccctgtagagtcctattcctctacctc
gtcgtgtaggtttccgtgtgggggaatgttgctcccatatcagaacagaagcaccttgcagggggaaggcttgtttccttaaggtctccgtatcctaaattaccacaaaggtgctgaaaatcagcaatcaaggcttgagttgcat
gaactgagctagttacactgcattacagtgactctgcagacaccactggacacagcaaagggccagaagacagcaagccttggccatagccgctgtcatcattcactccccacctcctaggagaaggaagatggaaacaag
tcaatgagttcataaaacagaaacccaggagtcattgcatcactccttatggctcagtggctcagattcactttataaaaacatactaatttcaatttcacatggtatgattcaaagagtcagaaattcatcgtctatcaagtggt
ctgaatgttatcctgcagaattttctctgaagatgaactggttcatccctagggctgtgcaatagtgtctcaatatccaatcactccccactatgtgaattaggccatatgttcttcaccggaacatttgaagaactcttgttgagat
tgtgcagttttagacactgggtcatcaagataaaagacacagaagctctgagaacacacaacttattttcacttgtccaaaataatcatcggctagtaagcttttattcattcatttttaaattgtcattgaaaaatgtatcaactt
agttttagaggaaaatcagtcatgtttgttaaattgatcatctacttgaaaactgtctttctggaaagaagaaaagatgttgctctctgtgcctgcatcctttggtgaggcctggagatgacctgggaaaaacacttggtgaccatt
accctggtagataagtagcatggcgggttaatcattaactacaaggaacccctagtgatggagttggccactccctctctgcgcgctcgctcgctcactgaggccgggcgaccaaaggtcgcccgacgcccgggctttgcccgg
gcggcctcagtgagcgagcgagcgcgcag

c

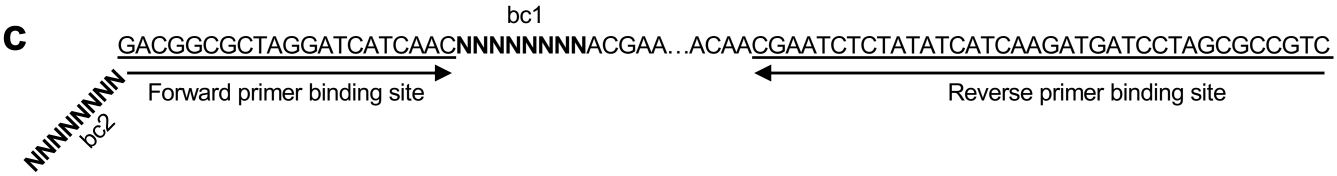

S1 Fig. Barcoded construct and PCR design. (a) Schematics showing the barcoded AAV construct, not drawn to scale. The first barcode (bc1) in the TuD and the second barcode (bc2) in the forward PCR primer are rainbow-colored. Note that each unique bc1 was used to pair with a unique capsid variant during rAAV production, and that bc2 was used in PCR to differentiate among tissue samples. **(b)** The DNA sequence of the construct shown in a. Key elements are color-coded as the scheme shown in a. Gray: ITR. Blue: stuffer. Green: U6 promoter. Yellow: TuD. NNNNNNNN: bc1. **(c)** Binding sites of the forward and reverse PCR primers shown in a. Binding sites are underlined. Note that bc2 is included at the 5’ end of the forward primer.
