# Supplementary material for: A single amino acid variant in the variable region I of AAV capsid confers liver detargeting
Source: PLoS Pathog. 2025 Sep 17;21(9):e1013533. doi: 10.1371/journal.ppat.1013533 (PMC12456803; doi:10.1371/journal.ppat.1013533)
Supplement: S2 Fig — (a) Bar graph showing the count of Illumia sequencing (MiSeq) reads mapped to the unique vector transgene barcodes packaged in AAV8, AAV9, or AAV8 variants. Data were based on one biological repeat. (b) Scatter dot plot showing the relationship between the sequencing read counts by nanopore method (x-axis) and MiSeq method (y-axis). The linear regression statistics are shown. (PDF) [file ppat.1013533.s002.pdf]

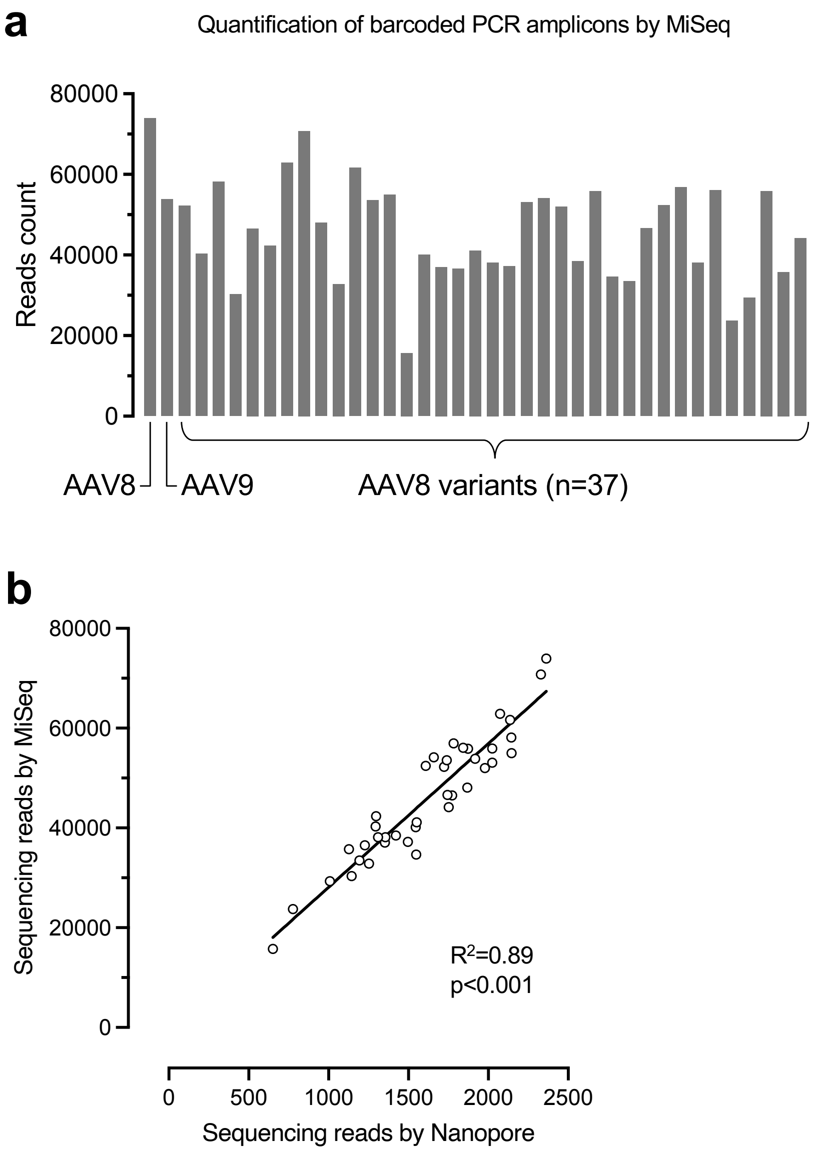

S2 Fig. Validation of the relative distribution of barcoded PCR amplicons derived from vector library DNA. (a) Bar graph showing the count of Illumia sequencing (MiSeq) reads mapped to the unique vector transgene barcodes packaged in AAV8, AAV9, or AAV8 variants. Data were based on one biological repeat. **(b)** Scatter dot plot showing the relationship between the sequencing read counts by nanopore method (x-axis) and MiSeq method (y-axis). The linear regression statistics are shown.
