# Supplementary material for: A single amino acid variant in the variable region I of AAV capsid confers liver detargeting
Source: PLoS Pathog. 2025 Sep 17;21(9):e1013533. doi: 10.1371/journal.ppat.1013533 (PMC12456803; doi:10.1371/journal.ppat.1013533)
Supplement: S3 Fig — Serum samples were collected immediately prior to rAAV injection (at rAAV injection) or immediately prior to euthanasia (at euthanasia). M: male. F: female. (PDF) [file ppat.1013533.s003.pdf]

	Ferret ID	Sex	At rAAV injection	At euthanasia
anti-AAV8	687721	M	<1:5	1:320
	687741	M	<1:5	1:320
	678861	F	<1:5	1:640
anti-AAV9	687721	M	<1:5	1:640
	687741	M	<1:5	1:640
	678861	F	<1:5	1:640

S3 Fig. Tabulation of neutralizing antibody titers in ferret serum samples. Serum samples were collected immediately prior to rAAV injection (at rAAV injection) or immediately prior to euthanasia (at euthanasia). M: male. F: female.
