# Supplementary material for: A single amino acid variant in the variable region I of AAV capsid confers liver detargeting
Source: PLoS Pathog. 2025 Sep 17;21(9):e1013533. doi: 10.1371/journal.ppat.1013533 (PMC12456803; doi:10.1371/journal.ppat.1013533)
Supplement: S4 Fig — Scatter dot plot showing the vector genome copy number per diploid host genome (vg/diploid genome) in the liver, heart, and tibialis anterior (TA) muscle collected from the mice (blue dots) and ferrets (green dots) treated with the vector library. Each dot represents an individual animal. Data are shown as mean and standard deviation. Statistical analysis is performed using two-tailed t-test. (PDF) [file ppat.1013533.s004.pdf]

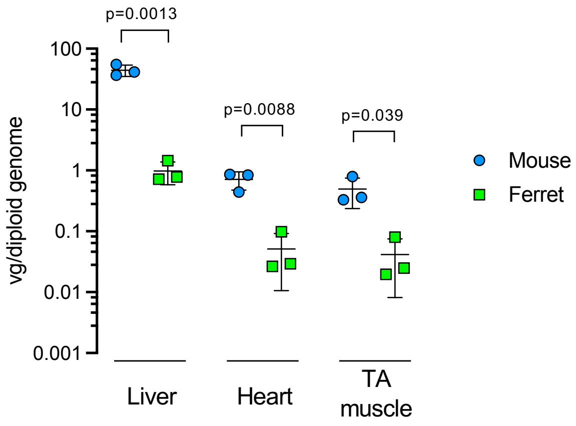

S4 Fig. Vector genome abundance in mouse and ferret tissues. Scatter dot plot showing the vector genome copy number per diploid host genome (vg/diploid genome) in the liver, heart, and tibialis anterior (TA) muscle collected from the mice (blue dots) and ferrets (green dots) treated with the vector library. Each dot represents an individual animal. Data are shown as mean and standard deviation. Statistical analysis is performed using two-tailed t-test.
