# Supplementary figures and images for: A single amino acid variant in the variable region I of AAV capsid confers liver detargeting
Source: PLoS Pathog. 2025 Sep 17;21(9):e1013533. doi: 10.1371/journal.ppat.1013533 (PMC12456803; doi:10.1371/journal.ppat.1013533)

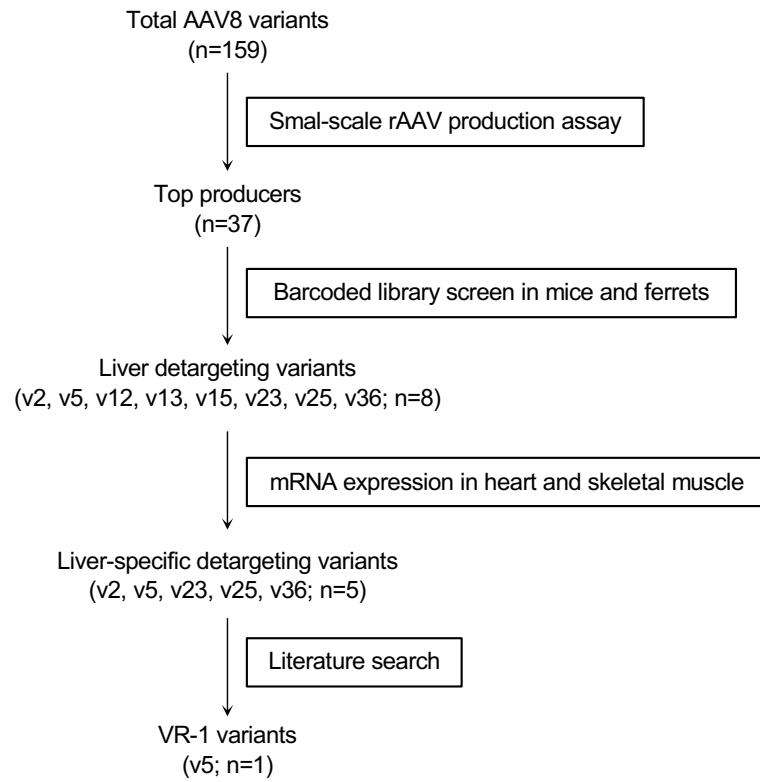

**S5 Fig. Workflow to select AAV8 variants.**

Supplement: S5 Fig — (PDF) [file ppat.1013533.s005.pdf]
