# Supplementary material for: A single amino acid variant in the variable region I of AAV capsid confers liver detargeting
Source: PLoS Pathog. 2025 Sep 17;21(9):e1013533. doi: 10.1371/journal.ppat.1013533 (PMC12456803; doi:10.1371/journal.ppat.1013533)
Supplement: S6 Fig — The parental capsid of each vector pair is indicated below the bars. The mutant capsids are AAV8.N271 in the AAV8 pair, AAV9.N270D in the AAV9 pair, and MyoAAV.N270D in the MyoAAV pair. All vectors carry the same vector genome that expresses EGFP. (PDF) [file ppat.1013533.s006.pdf]

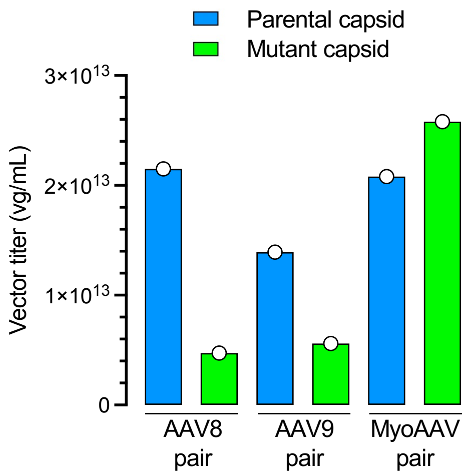

S6 Fig. Vector titers by large-scale production. The parental capsid of each vector pair is indicated below the bars. The mutant capsids are AAV8.N271 in the AAV8 pair, AAV9.N270D in the AAV9 pair, and MyoAAV.N270D in the MyoAAV pair. All vectors carry the same vector genome that expresses EGFP.
