# Supplementary material for: A single amino acid variant in the variable region I of AAV capsid confers liver detargeting
Source: PLoS Pathog. 2025 Sep 17;21(9):e1013533. doi: 10.1371/journal.ppat.1013533 (PMC12456803; doi:10.1371/journal.ppat.1013533)
Supplement: S7 Fig — (a-c) EGFP (green) and GAPDH (red) signals in the liver, heart, and tibialis anterior (TA) muscle tissue lysates. Mice were treated with AAV8 or AAV8.N271D vectors (a), AAV9 or AAV9.N270D vectors (b), and MyoAAV or MyoAAV.N270D vectors (c). Mice treated with PBS serve as negative controls. Different treatment groups are separated by dashed white lines to enhance visualization. Each lane represents an individual mouse. (d) Comparison among AAV8, AAV9, and MyoAAV.N270D vectors by running relevant samples on the same gels. Quantification is shown on the right side. Each dot represents an individual mouse. The box extends from the first to the third quartiles with the line inside denoting median. The whiskers end at minimum and maximum values. The fold changes of medians and p values are labeled. Statistical analysis is performed using two-tailed non-parametric Mann-Whitney U test. (PDF) [file ppat.1013533.s007.pdf]

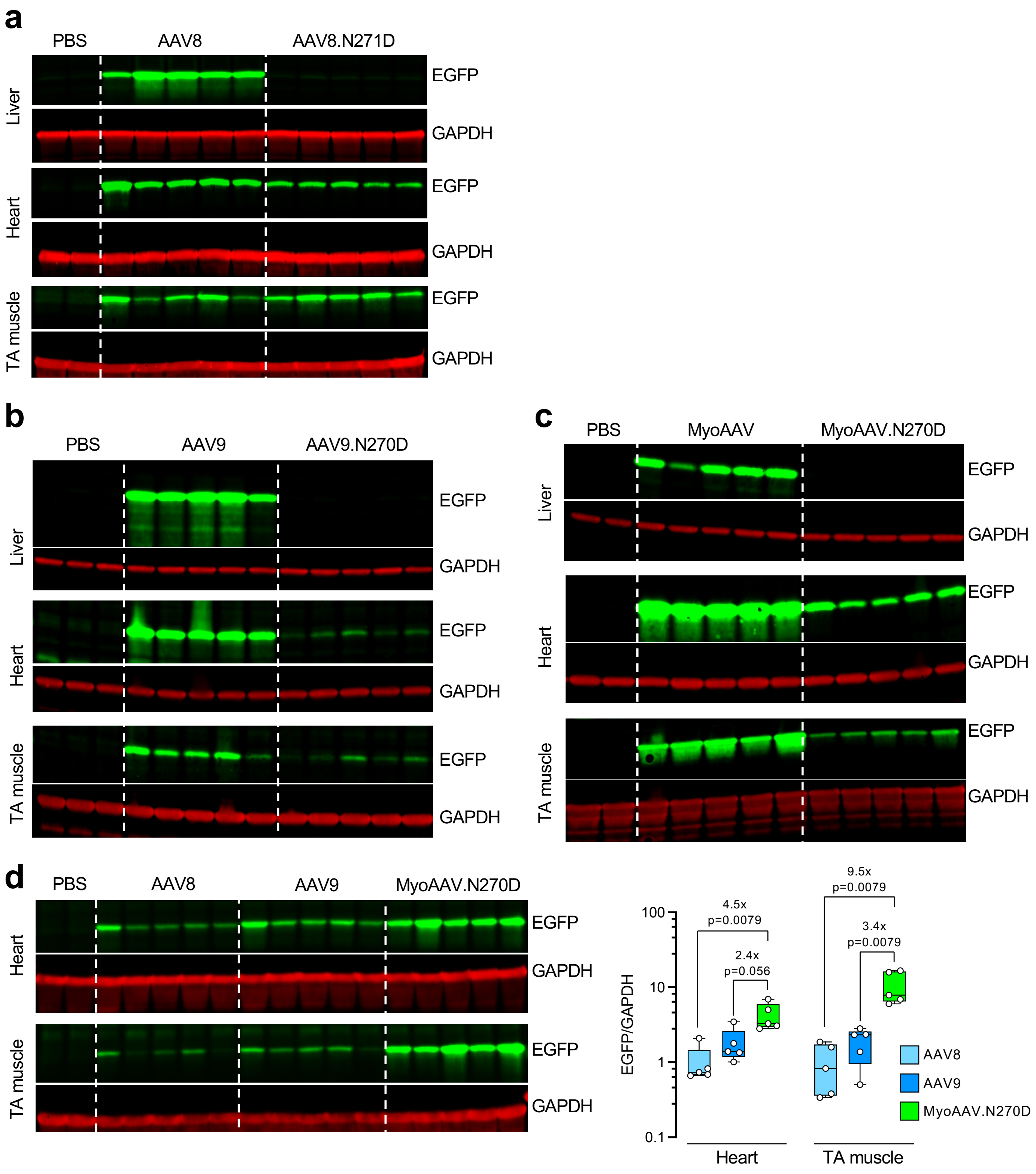

S7 Fig. Western blotting to quantify transgene expression. (a-c) EGFP (green) and GAPDH (red) signals in the liver, heart, and tibialis anterior (TA) muscle tissue lysates. Mice were treated with AAV8 or AAV8.N271D vectors (a), AAV9 or AAV9.N270D vectors (b), and MyoAAV or MyoAAV.N270D vectors (c). Mice treated with PBS serve as negative controls. Different treatment groups are separated by dashed white lines to enhance visualization. Each lane represents an individual mouse. **(d)** Comparison among AAV8, AAV9, and MyoAAV.N270D vectors by running all relevant samples on the same gels. Quantification is shown on the right side. Each dot represents an individual mouse. The box extends from the first to the third quartiles with the line inside denoting median. The whiskers end at minimum and maximum values. The fold changes of medians and p values are labeled. Statistical analysis is performed using two-tailed non-parametric Mann-Whitney U test.
