# Supplementary material for: A single amino acid variant in the variable region I of AAV capsid confers liver detargeting
Source: PLoS Pathog. 2025 Sep 17;21(9):e1013533. doi: 10.1371/journal.ppat.1013533 (PMC12456803; doi:10.1371/journal.ppat.1013533)
Supplement: S8 Fig — The levels in liver relative to those in heart (a) and TA muscle (b) in individual mice are calculated using the data shown in Fig 4 and plotted. Each dot represents an individual mouse. The box extends from the first to the third quartiles with the line inside denoting median. The whiskers end at minimum and maximum values. The fold changes of medians and p values are labeled. Statistical analysis is performed using two-tailed non-parametric Mann-Whitney U test. (PDF) [file ppat.1013533.s008.pdf]

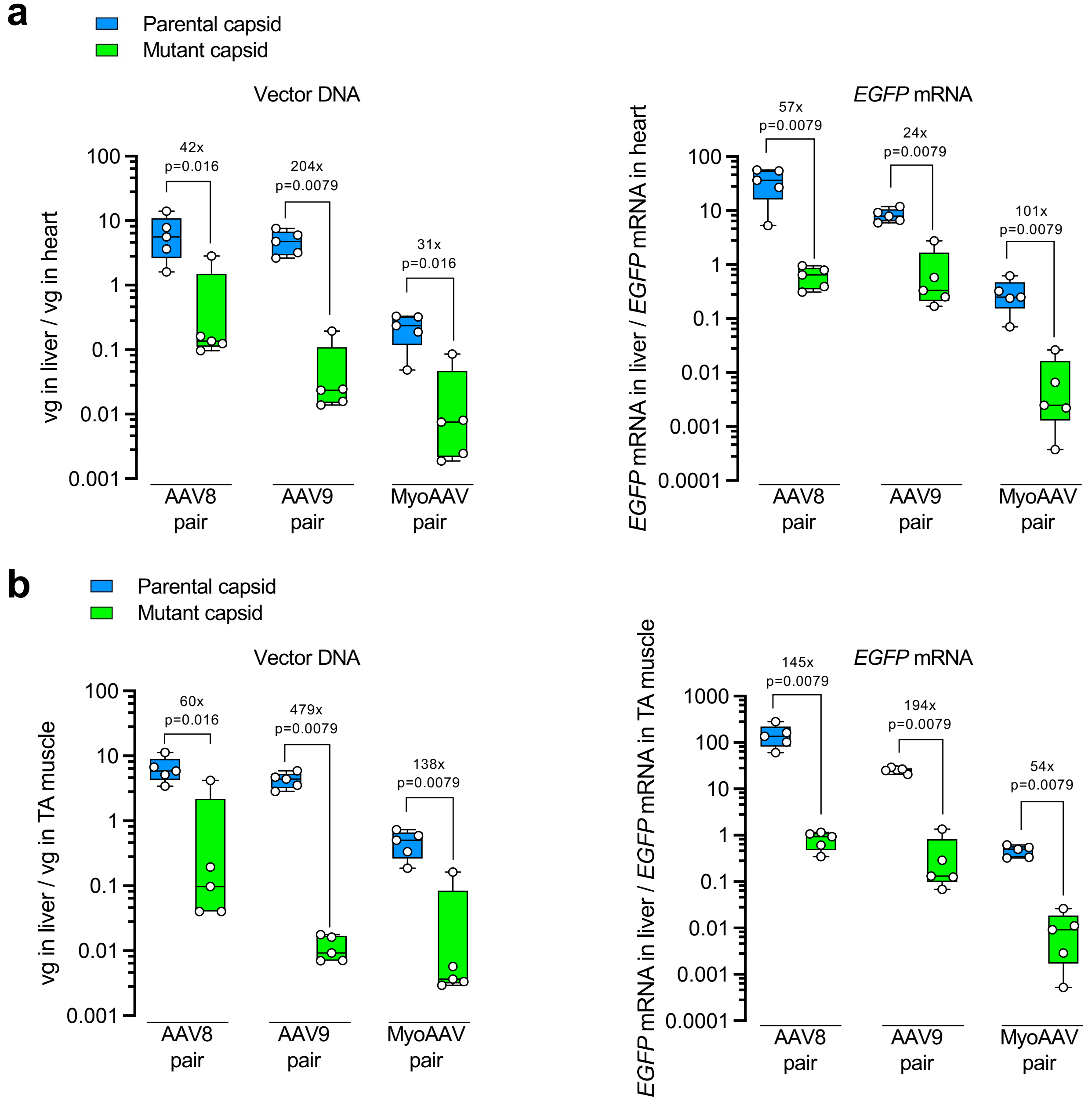

S8 Fig. Relative gene delivery and transgene expression. The levels in liver relative to those in heart (a) and TA muscle (b) in individual mice are calculated using the data shown in Figure 4 and plotted. Each dot represents an individual mouse. The box extends from the first to the third quartiles with the line inside denoting median. The whiskers end at minimum and maximum values. The fold changes of medians and p values are labeled. Statistical analysis is performed using two-tailed non-parametric Mann-Whitney test.
