# Supplementary material for: A single amino acid variant in the variable region I of AAV capsid confers liver detargeting
Source: PLoS Pathog. 2025 Sep 17;21(9):e1013533. doi: 10.1371/journal.ppat.1013533 (PMC12456803; doi:10.1371/journal.ppat.1013533)
Supplement: Attachment — Submitted filename: Human AAV8 library_R1_Point-by-point.docx [file ppat.1013533.s014.docx]

**Reviewers' Comments:**

Reviewer's Responses to Questions

**Part I - Summary**
Please use this section to discuss strengths/weaknesses of study, novelty/significance, general execution and scholarship.

Reviewer #1: Revision significantly improved the data description and explanation, which is easier to follow.

Reviewer #2: The authors have addressed all my previous concerns, hence there is nothing to add here.

Reviewer #3: Xing et al. revised the manuscript in response to the critiques raised by three reviewers. They were very responsive and most of the revisions seem appropriate. The data appear scientifically sound. That being said, the major concern that significantly diminishes the scientific merit of the paper remains unresolved, that is, whether the study findings will have a significant impact on the field. If the authors had identified transferable VR-I liver-detargeting mutations that could mediate transduction in non-hepatic target organs (e.g., heart and skeletal muscle) at levels equivalent to or higher than those of the parental AAV capsids, the study would likely warrant a high impact. However, the manuscript instead merely highlights a single, serendipitously identified mutation that reduces liver tropism while retaining the ability to transduce non-hepatic organs at moderately to substantially lower levels (4- to 16-fold) when introduced into capsids other than the one in which the phenotype was initially identified. Many similar liver-detargeting variants have already been reported in the literature, granted patents, and published patent applications. Importantly, the key claim that the liver-detargeted MyoAAV.N270D can mediate superior transduction in the heart and TA muscle compared to AAV8 and AAV9 (Lines 178-180) is not convincingly demonstrated by the data presented in the manuscript (Right panels in Fig. 4). Therefore, although the reported liver-detargeting mutation might be a useful addition to the growing list of such mutations, its discovery lacking a novel and compelling phenotype is unlikely to represent a significant advancement in the field.

**Response I-R1.R2.R3:** We thank all three reviewers for the positive feedback on our revision. We also appreciate Reviewer #3’s remaining concern about the reduced muscle transduction levels, especially when comparing MyoAAV.N270D to AAV8 and AAV9 (now lines 183-185). In the revised manuscript, we have provided additional experimental data and clarification to strengthen the key claim that MyoAAV.N270D outperformed AAV8 and AAV9 (please see details in Response II-R3-1 below).

**Part II – Major Issues: Key Experiments Required for Acceptance**
Please use this section to detail the key new experiments or modifications of existing experiments that should be absolutely required to validate study conclusions.

Generally, there should be no more than 3 such required experiments or major modifications for a "Major Revision" recommendation. If more than 3 experiments are necessary to validate the study conclusions, then you are encouraged to recommend "Reject".

Reviewer #1: all questions have been answered.

Reviewer #2: None

Reviewer #3: Lines 178-180, the authors’ argument, “Although MyoAAV.N270D.EGFP led to lower transduction levels in the heart and TA muscle as compared to MyoAAV.EGFP, it still outperformed AAV9.EGFP and AAV8.EGFP,” is not at all supported by the data presented. A cursory analysis for protein expression levels by eyes (Right panels in Fig. 4) revealed: MyoAAV.N270D=~0.1, AAV9=~1.0, and AAV8=~1.0 in the heart, and MyoAAV.N270D=~0.2, AAV9=~1.0, and AAV8=~1.0 in the TA muscle. This data does not align with the authors’ argument, and rather shows that AAV8 and AAV9 outperform MyoAAV.N270D. It appears that, to support their preferred claim, the authors focused only on mRNA expression levels while dismissing protein expression levels, which represent a more relevant and direct measure of transduction efficiency. In addition, the authors did not perform a statistical analysis to compare transduction levels in the heart and TA muscle between MyoAAV.N270D, AAV9, and AAV8 (i.e., a three-way comparison). The authors should analyze the data more rigorously and objectively to make a conclusion.

**Response II-R3-1:** We thank the reviewer for the thoughtful comment on comparing EGFP protein levels among AAV8, AAV9, and MyoAAV.N270D vectors. We apologize for not more clearly presenting and interpreting this dataset. The initial western blotting (WB) analysis (Supplementary Fig. 7a-c) was performed to compare each pair of parental and mutant capsids individually, and the values shown in the right panels in Fig. 4 reflect quantification relative to the parental capsid in each tissue. Because the AAV8, AAV9, and MyoAAV.N270D samples were run separately in different WB experiments, we were not able to directly compare the EGFP signals. We have now repeated WB to directly compare EGFP protein expression mediated by the AAV8, AAV9, and MyoAAV.N270D vectors. This analysis showed that MyoAAV.N270D.EGFP expressed the highest EGFP protein levels in the heart and TA muscle relative to AAV8.EGFP and AAV9.EGFP. We have now clarified the caveat of the original WB analysis, and included the new WB data in the revised manuscript **(lines 185-190, Supplementary Figure 7d)**.

Line 112, “with the potential caveat that different barcode sequences may introduce PCR bias.” This effect is often substantial. How did the authors address this caveat when analyzing and interpreting the data? This information is missing.

**Response II-R3-2:** We reasoned that using the same PCR method to quantify vector abundance in both the library and tissue samples would mitigate this potential bias, provided that library abundance is used as a normalization control. We have now revised the manuscript to include this information **(lines 113-115)**.

Please add a "Statistical analysis" paragraph in the Materials and Methods section describing the statistical methods used for data comparisons. In addition, in each figure legend, please describe briefly how the data were statistically analyzed, including the tests performed and any corrections made for multiple comparisons, if applicable. Moreover, please indicate which type of test, one-tailed or two-tailed, was used.

**Response II-R3-3:** We thank the reviewer for suggesting the inclusion of additional details on the statistical analysis. We have revised the Materials and Methods section **(lines 361-365)** and relevant figure legends accordingly.

**Part III – Minor Issues: Editorial and Data Presentation Modifications**
Please use this section for editorial suggestions as well as relatively minor modifications of existing data that would enhance clarity.

Reviewer #1: Fig1 a: tittle for x axis is suggested to be added to facilitate the data interpretation. Please state how AAV yield from each variant was compared and how comparisons were made to ensure fairness? Was any internal control used to normalize transfection efficiency or yield? It is noteworthy that certain mutations result in a dramatic decrease in yield—additional explanation or hypotheses would be valuable. Was the mutation in v2, 5, 12, 15, 23, 25, 36 significantly effected in yield with AAV8, AAV9 and Myo?

**Response III-R1-1:** We thank the reviewer for the suggestions to improve clarity regarding rAAV production and titers. (1) We have modified Fig. 1a to include a title for x axis. (2) We have added a description of the measures taken to carefully compare rAAV productivity, including details on cell culture condition and normalization control **(lines 263-267)**. (3) We have revised the manuscript to provide two potential reasons underlying the reduced titers **(lines 98-99)**. (4) We produced AAV8, AAV9 and MyoAAV only harboring the v5 mutation. As expected from the small-scale rAAV production assay, the large-scale production also showed that the mutant capsid compromised rAAV titers as compared with the respective parental capsid. We have now included this data **(lines 162-164; Supplementary Figure 6)**.

Line124: Please specify the method used to generate the pooled AAV preparation from single mutation and how the viral genome (vg) titer was calculated to achieve the final dosing concentration of 2 × 10¹³ vg/kg for injection.

**Response III-R1-2:** We have now included details on rAAV library preparation in the Materials and Methods section **(lines 279-284)**.

Line 152: Among so many interesting single mutation variants, N271D was selected for further study. Please rephrase this sentence to explicitly state the rationale for a smooth transition in the context.

**Response III-R1-3:** We have revised the manuscript to clarify the rationale and improve the transition **(lines 154-155)**.

Discussion: It would strengthen the discussion to further elaborate on how liver de-targeting by the N271D mutation appears to be independent of AAV serotype, as similar trends are observed across AAV8, AAV9, and Myo capsids. Expanding on this point would provide insight beyond the role of capsid deamidation alone.

**Response III-R1-4:** We agree with the reviewer that focusing solely on capsid deamidation may be overly narrow and misleading. We have revised the manuscript to also discuss the potential impact of the N271D mutation on cross-capsid receptor binding as a plausible alternative mechanism **(lines 211-213)**.

Reviewer #2: None

Reviewer #3: Lines 59-60. The authors are encouraged to double-check whether the statement, “Indeed, VR-1 contributes to the 3-fold protrusions on AAV capsid”, is appropriate.
**Response III-R3-1:** We apologize for not including a proper reference for this statement (now lines 59-60). In the revised manuscript, we have added a reference that maps all VRs to the AAV capsid by color coding (Figure 2 in ref #12). It shows that VR-1 is a part of the 3-fold protrusions.

Fig. 1a. To improve clarify, please provide information on how each point corresponds to the AAV8 variants listed in Table S1.

**Response III-R3-2:** We have added this information in Table S1 (“Rank in Fig. 1a” column).

Lines 135-136, the statement, “low AAV vector DNA abundance in tissues does not necessarily result in low functional transduction,” is misleading. This phenomenon is more relevant when an organ carries a relatively high AAV vector genome copy number but exhibits disproportionately low transgene expression, rather than the opposite scenario, where vector genome copy number is low but transgene expression is disproportionately high.

**Response III-R3-3:** We agree with the reviewer and have revised the language to be more straightforward and intuitive **(lines 137-139)**.

Line 180, “middles” is a typo. It should be “middle.”

**Response III-R3-4:** We apologize for the typo, which has been corrected.

Lines 217-219, regarding the following statement, "The dramatic liver detargeting phenotype of the AAV8.N271D, AAV9.N270D, and MyoAAV.N270D vectors is accompanied by moderate reductions in targeting the heart and TA muscle (Figure 4)." Since AAV9.N270D exhibits 15- to 16-fold (substantial) reduction in transgene expression in the heart, "moderate" in the above statement is misleading. Please revise this statement appropriately (e.g., by removing AAV9.N270D).

**Response III-R3-5:** We thank the reviewer for the suggestion to improve accuracy, and have revised the manuscript accordingly **(line 229-231)**.

Lines 301-306. The authors need to provide a brief description explaining how raw read count numbers were converted to relative values presented in the figures, which is missing in the manuscript.

**Response III-R3-6:** We have revised the manuscript to explain how the relative values were calculated **(lines 329-332)**.

Line 400, "Mann Whitney test" should be "Mann-Whitney U test."

**Response III-R3-7:** We have made correction throughout the manuscript.

What is the justification for using a t-test for some datasets and a U test for others?

**Response III-R3-8:** We used t-test to compare means in a scatter plot and the Mann-Whitney U test to compare medians in a box plot. We have included this information in the revised manuscript **(lines 361-365)**.
